# Factors associated with lingual tonsil hypertrophy in Canadian adults

**DOI:** 10.1186/s40463-017-0209-z

**Published:** 2017-04-17

**Authors:** Matthew S. Harris, Brian W. Rotenberg, Kathryn Roth, Leigh J. Sowerby

**Affiliations:** 1 Department of Otolaryngology – Head & Neck Surgery, Schulich School of Medicine & Dentistry, Western University, London, ON Canada; 2 St. Joseph’s Healthcare, Western University, 268 Grosvenor Street, London, ON N6A 4 V2 Canada

**Keywords:** Laryngopharyngeal reflux, Reflux symptom index, Reflux finding score, Body mass index, Tonsillectomy, Lingual tonsil

## Abstract

**Background:**

Hypertrophy of the lingual tonsil tissue in the adult patient is thought to contribute to the pathophysiology of obstructive sleep apnea. The underlying etiology of lingual tonsil hypertrophy (LTH) in the adult patient is unclear and likely multifactorial. Previous studies have suggested that the lingual tonsils may undergo compensatory hyperplasia post-tonsillectomy in children, although it is unknown if this occurs or persists into adulthood. The purpose of this study was to determine what factors are associated with LTH in a population of Canadian adults.

**Methods:**

Adult patients presenting for consultation to an academic Rhinology/General Otolaryngology practice were eligible for enrollment. Demographic data including age, body mass index (BMI), Reflux Symptom Index (RSI), history of allergy, and history of tonsillectomy was collected via questionnaire. Endoscopic photographs of the base of tongue and larynx were captured. These were graded for LTH and Reflux Finding Scale (RFS) by blinded examiners. Statistical analysis was performed by comparing the mean LTH value to the variables of interest using two-tailed *T*-test. *P* < .05 was considered significant.

**Results:**

One hundred two subjects were enrolled. Age ranged from 18 to 78. 28 patients had previous tonsillectomy. This was not associated with a significant increase in lingual tonsil tissue (*r* = −0.05, *p* = 0.61). RFS >7 or RSI >13 was considered positive for laryngopharyngeal reflux. There was no difference in LTH based on RSI positivity (*p* = 0.44). RFS positivity correlated with increased lingual tonsil tissue (*p* < 0.05). BMI >30 was associated with increased lingual tonsil hypertrophy (*p* < 0.05).

**Conclusions:**

An elevated body mass index and positive Reflux Finding Score are associated with lingual tonsil hypertrophy in adults. Reflux symptom index, history of allergy and history of childhood tonsillectomy are not associated with LTH.

## Background

The lingual tonsils are composed of reactive lymphoid tissue at the base of the tongue. Hypertrophy of the lingual tonsils can present clinically as globus, dysphagia, and cause difficultly with exposure of the glottis during intubation. Lingual tonsil hypertrophy (LTH) can also contribute to obstructive sleep apnea (OSA) at the level of the oropharynx. In children, compensatory LTH has been observed after routine tonsillectomy [1]. Recently, Sung et al. examined factors that were associated with lingual tonsil hypertrophy in Korean patients with OSA. Obesity and endoscopic evidence of reflux were found to be associated with LTH [2]. More recently, Friedman et al. have further studied endoscopic examination of the lingual tonsils in order to standardize a grading scale [3].

To date, no study has examined with relationship with tonsillectomy as a child and LTH as an adult. There is also an emerging body of evidence that suggests environmental allergies may cause laryngeal symptoms, however, the symptom overlap and comorbidity between allergy and LTH has yet to be fully elucidated [4, 5]. The purpose of this study was to determine what factors are associated with LTH in a population of Canadian adults.

## Methods

Research ethics board approval was obtained at Western University (London, Ontario, Canada) for this project (HSREB # 104994). A prospective cross-sectional study enrolling consecutive patients presenting for routine consultation at an academic Rhinology and General Otolaryngology – Head & Neck Surgery practice was performed from March 2014 to June 2014. All patients older than 18 and requiring flexible nasopharyngoscopy as part of their physical examination were considered eligible for inclusion. Exclusion criteria included age less than 18, non-English speaking, illiteracy, and a history of previous sleep apnea surgery as an adult. Patients completed a questionnaire for demographic data, and completed the Reflux Symptom Index (RSI). An RSI of greater than 13 was considered positive for reflux [6]. Demographic factors examined included age, body mass index (BMI), history of diagnosed OSA, history of environmental allergies and history of childhood tonsillectomy. Tonsillectomy was considered to have been performed in childhood if the patient had tonsillectomy performed before the age of 18.

During routine nasopharyngoscopy, photographs of the base of tongue and larynx were captured for each patient. This was done with the patient in a standardized “sniffing” position with the tongue protruded. All photographic images were captured by a single surgeon and were standardized in distance from larynx and base of tongue. The nasopharyngoscope was positioned just past the soft palate and a photograph was taken with view of the base of tongue and posterior wall of the pharynx with the larynx centered in the photograph. Three blinded examiners reviewed the clinical photographs of the larynx and base of tongue in a randomized fashion. The blinded reviewers were all Fellows of the Royal College of Physicians and Surgeons of Canada in Otolaryngology with practices in general otolaryngology and rhinology, and none had sub-specialty interest or expertise in laryngology. The photographs of the larynx were evaluated to calculate the Reflux Finding Scale (RFS) score (Table 1) [6]. These were averaged to create a mean RFS score, which was considered positive if the score was greater than 7 [6]. The photographs of the base of tongue were graded for LTH based on the scale published by Sung (Table 2) [2]. Mean LTH values were subsequently also calculated for each patient.

**Table 1:** Reflux Finding Score developed by *Belafsky* et al. [4]

Subglottic Edema	2 = present
	0 = absent
Ventricular Obliteration	2 = partial
	4 = complete
Erythema/Hyperemia	2 = arytenoids only
	4 = diffuse
Vocal Fold Edema	1 = mild
	2 = moderate
	3 = severe
	4 = polypoid
Diffuse Laryngeal Edema	1 = mild
	2 = moderate
	3 = severe
	4 = obstructing
Posterior Commissure Hypertrophy	1 = mild
	2 = moderate
	3 = severe
	4 = obstructing
Granuloma/Granulation	2 = present
	0 = absent
Thick Endolaryngeal Mucous	2 = present
	0 = absent

**Table 2:** Lingual Tonsil Grading Scale used by *Sung* et al. [1]

Grade	Description
0	No lingual tonsil tissue
1	Spotted lingual tonsil tissue
	Base of tongue vasculature visible
2	Base of tongue vasculature obscured
	Vallecula visible
3	Vallecula obscured
4	Epiglottis obscured

Statistical analysis was performed by comparing the mean LTH value to the variables of interest using two-tailed *T*-test. These included BMI (>30 vs. <30), endoscopic evidence of reflux (RFS > 7 vs. RFS < 7), Reflux Symptom Index (RSI > 13 vs. RSI <13), and history of childhood tonsillectomy. Kolmogorov-Smirnov test confirmed normal distribution for age, BMI, RSI and mean RFS values. Fleiss’ Kappa for multiple observers was calculated for the grading of LTH and RFS scoring by the blinded reviewers. Linear regression was performed to determine if age correlated to lingual tonsil size.

## Results

One hundred and two patients were enrolled in the study (49 men, 53 women). Mean age was 48.0 years (19–78). Mean BMI was 28.2 (18.5–41.8). A previous diagnosis of gastroesophageal reflux disease or OSA was present in 20 (20%) and 15 (15%) participants respectively. Twenty-seven patients (26%) had a previous childhood tonsillectomy. Patient demographics are shown in Table 3.

**Table 3:** Patient Demographics

Demographic	Male	Female	Total
n	49	53	102
Age	46.7 (20–78)	49.1 (19–77)	48.0 (19–78)
BMI	28.6 (23.1–40.90)	27.8 (18.5–41.8)	28.2 (18.5–41.8)
Hx of Reflux	8 (16%)	12 (23%)	20 (20%)
Hx of Allergies	20 (41%)	28 (53%)	48 (47%)
Hx of OSA	11 (22%)	4 (8%)	15 (15%)
Hx of Childhood Tonsillectomy	15 (31%)	12 (23%)	27 (26%)

There was no correlation between age and lingual tonsil size (*r* = −0.05, *p* = 0.61). History of childhood tonsillectomy did not demonstrate a significant difference in LTH grading (*p* = 0.51). Patients with a BMI >30 had a significantly larger mean lingual tonsil grade than patients with BMI <30 (2.26 vs. 1.83, *p* = 0.035). Inter-rater agreement between blinded reviewers was graded as fair with Fleiss’ Kappa for both the Reflux Finding Score (κ = 0.34) and lingual tonsil hypertrophy grade (κ = 0.41). A positive Reflux Finding Score was also associated with significant increase in mean lingual tonsil grade (2.31 vs. 1.76, *p* = 0.008). A positive Reflux Symptom Index showed no significant difference in lingual tonsil grading (*p* = 0.44). History of allergy also failed to demonstrate a significant difference in lingual tonsil grading (1.86 vs. 1.97, *p* = 0.52). Results are summarized in Table 4.

**Table 4:** Results of Two Tailed T-tests

Variable	BMI <30	BMI >30	BMI	Lingual Tonsil Grade
	*n* = 73	*n* = 29		
Mean Age	47.4	49.8	<30	1.83 ± 0.85
Tonsillectomy	19 (26%)	8 (28%)	>30	2.26 ± 0.85
RSI	13.5	15.2		*p* = 0.035
RFS	6.3	6.0		
LTH	1.83	2.26		
Variable	RFS <7	RFS >7	RFS	Lingual Tonsil Grade
	*n* = 58	*n* = 44		
Mean Age	45.1	52.1	<7	1.76 ± 0.82
Tonsillectomy	20 (34%)	7 (16%)	>7	2.31 ± 0.85
BMI	28.7	27.7		*p* = 0.008
RSI	14.2	14.4		
LTH	1.76	2.31		
Variable	RS1 < 13	RSI >13	RSI	Lingual Tonsil Grade
	*n* = 56	*n* = 46		
Mean Age	45.3	50.6	<13	1.99 ± 0.84
Tonsillectomy	15 (27%)	12 (26%)	>13	1.95 ± 0.90
BMI	28.0	28.6		*p* = 0.44
RFS	6.3	6.2		
LTH	1.99	1.95		
Variable	No Tonsillectomy	Previous Tonsillectomy	Palatine Tonsils	Lingual Tonsil Grade
	*n* = 75	*n* = 27		
Mean Age	44.7	56.5	Yes	2.08 ± 0.78
BMI	28.8	28.1	No	1.93 ± .91
RSI	12.8	14.2		*p* = 0.51
RFS	6.5	6.1		
LTH	2.08	1.93		
Variable	Allergy	No Allergy	Allergy	Lingual Tonsil Grade
	*n* = 29	*n* = 73		
Mean Age	44.2	49.5	Yes	1.86 ± 0.73
BMI	28.4	28.1	No	1.97 ± 0.75
RSI	15.6	12.2		*p* = 0.035
RFS	6.3	5.8		
LTH	1.86	1.97		

## Discussion

Lingual tonsil hypertrophy can play a major role in OSA and in difficult intubations, yet little attention has been paid to the etiology. There appears to be a complex interplay with laryngopharyngeal reflux (LPR) emerging as a strong potential contributor to LTH and, subsequently, OSA. Previous authors have also demonstrated an association between LTH and OSA, BMI, age and smoking, but there has not been general agreement [1, 7]. This study adds further support to an association between a positive reflux finding score and BMI with lingual tonsil hypertrophy, but does not support a history of childhood tonsillectomy or age being associated with lingual tonsil size in adults.

Sung et al. demonstrated a correlation between BMI, reflux finding score and lingual tonsil hypertrophy in OSA patients [1]. A trend was also seen for a negative correlation with age, but was not statistically significant. This association has also been supported by Friedman’s group in Chicago, where a statistically significant association was found between increasing LTH and decreasing age, RSI >10 and positive smoking status [7]. Interestingly, they did not, however, find an association with BMI, PPI use or allergy medication use. Our study failed to identify any trend or correlation between age and LTH. Part of the discrepancy may be related to the scoring scale used by the respective authors. In this study, the LTH scoring scale described by Sung et al. was used because Friedman’s scale was not yet published at the time of study [8]. Previous methods of measurement using both CT and MRI have also been described, but given the expense are not justified for routine evaluation in a universal-payer system such as in Canada [1]. Both Sung and Friedman’s scales have demonstrated similarly good inter-rater agreement. The kappa for Sung’s grading scale was reported to be 0.73, while Friedman’s grading scale had a reported kappa of 0.78 for the video assessment and 0.87 for live assessment. An advantage of Friedman’s scale is the use of video and various positions of the tongue, which appears to allow for greater inter-rater agreement and consistency and may be a source of some of our inter-rater disagreement. Sung’s group did, however, demonstrate good correlation with measurements on MRI suggesting validity of their scale and the use of standardized photography for grading [1]. Lastly, although lingual tonsil hypertrophy has been observed in pediatric patients with prior adenotonsillectomy, a post-mortem study examining 497 corpses found only 16 (3.2%) had LTH. Of those, 6 (37.5%) had evidence of previous tonsillectomy versus 119 (23.9%) of the whole study sample, but formal statistical analysis was not performed [9].

It is likely there is a complex interplay between obesity, OSA, LPR and LTH. DelGaudio et al. demonstrated increasing severity of LTH with more severe nasopharyngeal reflux on PH probe testing, but also found that those with mild LTH had a BMI that was 8 points lower than those in the moderate and severe groups. They did not assess for OSA in the studied population [10]. It is known that OSA creates negative intra-thoracic pressure that can exacerbate reflux, but other evidence suggests that a vasovagal reflex arc may be triggered by refluxate [11]. Two previous studies have demonstrated that treatment of reflux can help in the treatment of OSA. Friedman demonstrated an average reduction in AHI from 38 to 29 in patients with a negative pH study after treatment with proton pump therapy [12]. Senior found that the apnea index decreased by 31% and respiratory disturbance index decreased by 25% with treatment with omeprazole and lifestyle modifications after one month of therapy in patients with confirmed reflux on pH probe testing [13].

Limitations of this study include the cross-sectional nature of the study and lack of confirmation of OSA or LPR by objective testing. A recent study by Chang et al. examining the reliability of the RFS score among general otolaryngologists found only fair agreement and would suggest it is not reliable among non-expert users [14]. This has been the case with other studies as well [4]. Unfortunately, a more reliable endoscopic grading tool does not yet exist and use of it may have contributed to error in this study. The sample size of this study also potentially risks a type II error but no trend was present in the tonsillectomy and LTH data, making this unlikely. The *p*-value of the previously mentioned cadaveric study examining tonsillectomy and LTH was calculated using Fisher’s exact test to be 0.25, with a sample size of almost 500 [9]. With no previously published studies finding an association between adult LTH and childhood tonsillectomy, establishing the required sample size a priori was not possible. We performed a sample size calculation assuming that tonsillectomy would produce an LTH difference similar to what was seen in this study with the RFS and BMI. This gave a sample size of 68 with a beta of 0.8. A further refinement of this study would have involved collecting specific data regarding the age at which childhood tonsillectomy was performed.

## Conclusion

Our study is in agreement with previous studies that have demonstrated a correlation between obesity, endoscopic evidence of reflux and lingual tonsil hypertrophy. There does not appear to be a relationship between lingual tonsil hypertrophy and a history of previous childhood tonsillectomy.

## Abbreviations

BMI: Body mass index
HSREB: Health sciences research ethics board
LPR: Laryngopharyngeal reflux
LTH: lingual tonsil hypertrophy
OSA: Obstructive sleep apnea
RFS: Reflux finding score
RSI: Reflux symptom index
